# CRISPR-Mediated Gene Activation (CRISPRa) of pp38/pp24 Orchestrates Events Triggering Lytic Infection in Marek’s Disease Virus-Transformed Cell Lines

**DOI:** 10.3390/microorganisms9081681

**Published:** 2021-08-08

**Authors:** Poornima Roy, Katy Moffat, Venugopal Nair, Yongxiu Yao

**Affiliations:** 1 Viral Oncogenesis Group, The Pirbright Institute, Pirbright, Surrey GU24 0NF, UK; poornimamurthyin@yahoo.com (P.R.); kathryn.moffat@pirbright.ac.uk (K.M.); 2 The Jenner Institute Laboratories, University of Oxford, Oxford OX3 7DQ, UK; 3 Department of Zoology, University of Oxford, Oxford OX1 3SZ, UK

**Keywords:** CRISPRa, pp38, latent/lytic switch, apoptosis

## Abstract

Marek’s disease (MD) is an immunosuppressive and highly contagious lymphoproliferative disease caused by Marek’s disease virus (MDV) in poultry. Lymphoblastoid cell lines (LCLs) generated ex vivo from MD lymphomas are considered excellent models to study virus-host molecular interactions. LCLs mostly have latently infected MDV genome, but many of them also have varying populations of lytically-infected cells, thus making them very suitable to examine the molecular events associated with the switch from latent to lytic infection. MDV-encoded phosphoprotein 38 (pp38) is readily detectable in lytically-infected LCLs and hence considered as a biomarker for lytic infection. Whilst previous studies have suggested that pp38 is essential for the early cytolytic infection of B-cells, its role in the switch from latent to lytic infection of LCLs is still unclear. pp24, another phosphorylated protein in the same protein complex, shares the same promoter and N-terminal 65 amino acids as pp38. In this study we employed CRISPR activation (CRISPRa) technology for targeted activation of pp38/pp24 in LCLs to investigate their role in inducing lytic infection. Our results show that enforced expression of pp38/pp24 through CRISPRa induces orchestrated upregulation of other MDV genes including ICP4, gB, Meq and pp14 as well as differential expression of host genes thereby facilitating lytic infection. Our results also show that pp38/pp24 expression induces the lytic switch through inhibiting apoptosis.

## 1. Introduction

Marek’s disease (MD) is caused by Marek’s disease virus (MDV), a cell-associated, highly oncogenic alphaherpesvirus belonging to the genus Mardivirus [1]. MD is characterized by rapid-onset T-cell lymphomas, immunosuppression and paralysis in poultry [2]. MDV pathogenesis in the chicken host is complex involving early lytic infection followed by latent infection leading to neoplastic transformation of CD4+ T-cells resulting in lymphomas of multiple visceral organs. Latent and lytic infections are the two main states of the disease where MDV has distinct interactions with the host-cell machinery. Latency is the quiescent state where the virus limits its gene expression helping it to escape the onslaught of the host immune response. During the lytic stage of infection, the virus is in an active state overriding the various restrictive factors such as epigenetic regulation (DNA methylation, histone modification) [3], host cell niche [4] and expression of viral latency-associated transcripts (LATs) [5,6]. The lytic stage of MDV replication has a tightly regulated gene expression profile with distinct phases of expression of immediate-early (e.g., ICP4), early (e.g., pp38) and late genes (e.g., gB) [7].

While the majority of the tumour cells are latently infected with limited expression of viral genes, a significant number of cells in the lymphoma also undergo lytic infection and cell death with active expression of multiple viral genes. As clonal populations of transformed tumour cells derived from primary MD lymphomas, lymphoblastoid cell lines (LCLs) are valuable resources to study virus-host interactions in transformed cells. Many LCLs contain populations of cells that constantly undergo a switch from latency to lytic infection and hence are excellent tools for studying events involved in the switch to lytic infection. Parcells et al. (1999) demonstrated that LCLs derived from tumours induced by recombinant MDV-expressing lacZ marker showed expression of lytic viral genes pp38, US1, gB, gI and US10 when treated with 5′-iododeoxyuridine [8]. Lytic replication could be induced in LCLs using chemical inducers such as HDAC inhibitor sodium butyrate (NaB) or methylation inhibitor 5-azacytidine (5-Aza-CR), demonstrating that epigenetic modifications are involved in maintaining latency [3]. Differential expression of several genes, including upregulation of 82 viral genes such as pp38, ICP4, gE and gI, was identified in populations undergoing a spontaneous switch to lytic infection in LCL derived from lymphomas induced by recombinant pRB1B-UL47eGFP virus [4].

Because of the rapid induction during lytic infection from an almost complete absence of expression during the latent phase, MDV-encoded phosphoproteins pp38 and pp24 are widely considered as biomarkers for lytic switch of infection [9]. pp38 and pp24, which share the 65 N-terminal amino acids, are encoded from genes located at the junction of the unique long (U_L_) and the repeat long (R_L_) regions of the MDV genome and flanked with an MDV origin of lytic replication (OriLyt) [10,11,12]. Transcription of pp38 and pp24 genes is regulated by the bidirectional promoter-enhancer of the 1.8 kb gene family [13]. Previous studies have shown that pp38 is essential for the lytic infection of B-cells, while it was dispensable for lytic infection of the epithelial cells of feather follicles or the induction of tumours [14]. A recent study has also demonstrated that pp38/pp24 can be deleted from LCLs without affecting MDV latency or cell proliferation, although this study did not examine the ability of the mutant LCL to switch to lytic infection [15]. While global epigenetic modifiers such as HDAC and DNA methylase inhibitors can be used for initiating lytic infection, these treatments have broader effects on the cell and therefore are not useful to examine the direct role of pp38/pp24 in the lytic switch. We have employed a robust CRISPR activation (CRISPRa) strategy for precise targeted activation of pp38/pp24 expression in LCLs to examine its direct role in the switch to lytic infection.

Advances in CRISPR/Cas9 technology have revolutionized functional investigations of gene expression [16,17]. Many improved techniques, adapted from the CRISPR/Cas9 system, have been developed to regulate the gene expression at the transcriptional levels such as CRISPRa and CRISPRi (CRISPR interference) by fusing or otherwise recruiting the appropriate transcriptional effector domains to the nuclease-deactivated Cas9 or dead Cas9 (dCas9) [18]. The CRISPRa system, utilising dCas9 fused to transcriptional activators that are guided to bind to a specific promoter by guide RNAs for targeted activation of gene expression, is increasingly used to study gene functions. For example, robust upregulation of FOXP3 was achieved with CRISPRa through targeting of the core promoter in HEK293 and Jurkat T cell lines, representing a potential approach to increase the pool of Tregs within the lymphocyte population [19]. In addition to targeting cellular genes, the CRISPRa system has also been used to trigger the Kaposi’s sarcoma-associated herpesvirus (KSHV) reactivation in latently infected cells by selectively activating the ORF50 gene directly from the virus genome [20]. In our present study, we employed the CRISPRa system to activate pp38/pp24 expression in LCL 4523T cells to investigate its role in the induction of MDV lytic replication. The upregulation of other MDV lytic genes, differential expression of host genes and increased MDV reactivation of 4523T in chicken embryo fibroblast (CEF) cells indicated that pp38/pp24 induces lytic replication in MDV transformed LCLs.

## 2. Materials and Methods

### 2.1. Cell Culture

CD4+ MDV-transformed LCLs 4523T from a testes lymphoma, 760S from a spleen lymphoma and 226O from an ovary lymphoma induced by the RB-1B strain of MDV and HP8 from a GA strain-induced tumour were cultured at 38.5 °C in 5% CO2 in RPMI 1640 medium (Sigma-Aldrich, Burlington, MA, USA) containing 10% fetal calf serum (Sigma-Aldrich, Burlington, MA, USA) along with 2% chicken serum and 10% tryptose phosphate broth (Sigma-Aldrich, Burlington, MA, USA), as described previously [21]. CEF cells were isolated from 10-day-old SPF chicken embryos (VALO BioMedia GmbH, Osterholz-Scharmbeck, Germany). Cells were cultured in M199 medium (Life Technologies, Paisley, UK), supplemented with 5% fetal bovine serum (FBS, Sigma, Dorset, UK), 100 units/mL of penicillin and streptomycin (GIBCO, Waltham, MA, USA), 0.25 µg/mL Fungizone (Sigma-Aldrich, Burlington, MA, USA) and 10% tryptose phosphate broth (GIBCO, Waltham, MA, USA) [22].

### 2.2. Generation of dCas9-VP64 Stable Cell Line

The pHAGE TRE dCas9-VP64 lentiviral expression vector (Addgene) was electroporated into 4523T cells using NEPA21 Electroporator as described previously [23]. Briefly, 1.2 × 10^6^ of 4523T cells were resuspended in 96 μL Opti-MEM medium (Thermo Fisher Scientific, Waltham, MA, USA) and mixed with 10 μg of pHAGE TRE dCas9-VP64 in 4 μL Opti-MEM to make a total volume of 100 μL. The cells were then electroporated with optimized conditions at 275 V and a pulse width of 1.5 ms of poring pulse. The transfected cells were selected with geneticin (Sigma-Aldrich, Burlington, MA, USA) 48 h (h) post electroporation at a concentration of 0.45 µg/mL. Geneticin selected cells were then single cell sorted using a fluorescent activated cell sorter (FACS), the FACSAria U3 (BD Bioscience, Wokingham, Berkshire, UK). The positive single cell clones were identified via PCR using forward primer 5′-CCTGAATGCAGTGGTAGGCA-3′ and reverse primer 5′-CATTCGTTTCCGGCCGTTTT-3′ from the dCas9-VP64 sequence. For all further studies, clone 4 of 4523T-dCas9-VP64 (C4) cells was used.

### 2.3. gRNA Design

The gRNA designing tool (http://crispor.org, CRISPOR 4.8, accessed on 4 August 2019) was used for gRNA design. The lack of good quality gRNAs in the promoter region led us to investigate potential gRNAs in the 5′-UTR region of pp38/pp24. Two gRNAs with the highest efficiency and low off-target scores, located in the 5′-UTR, were chosen. The sequences 5′-TTCCAAACGATTAACAACCGGGG-3′ (forward strand, position −283 to −265 relative to pp38/pp24 gene ORF) for gRNA-1 and 5′-GTCCCCGGTTGTTAATCGTTTGG-3′ (reverse strand, position −278 to −259 relative to pp38/pp24 gene ORF) for gRNA-2 were synthesised using a two-part guide RNA system containing crRNA-tracrRNA guide complex from Integrated DNA Technologies (IDT, Diego, CA, USA). The 36-mer crRNA contains the gene-specific 20-nt target sequence followed by the 16-nt sequence that base-pairs with the tracrRNA. The 67-mer tracrRNA contains the gRNA-scaffold sequence as well as the 16-nt sequence complementary to crRNA. The lyophilized crRNA and tracrRNA pellets were resuspended in Duplex buffer (IDT) at 200 μM and stored in aliquots at −80 °C. A non-specific gRNA control, sgA [24], was chemically synthesised and used for transfection in the same way.

### 2.4. Transfection of gRNAs into 4523T-dCas9-VP64 Cells

To form the crRNA and tracrRNA complex, equimolar concentrations of crRNA and tracrRNA were mixed in an RNAse-free Eppendorf tube, heated at 95 °C for 5 min and left at room temperature for 10 min. The crRNA-tracRNA complex of either two pp38/pp24 gRNAs or sgA was electroporated into C4 cells, as described in Section 2.2. As pHAGE TRE dCas9-VP64 plasmid contains a tetracycline response element, 1 μg/mL of doxycycline (Dox) was added to the transfected cells for the dCas9-VP64 activation. At 24 h post transfection (pt), cells were harvested for RNA extraction and plaque assay.

### 2.5. FACS Analysis

To analyze the pp38/pp24 protein expression following CRISPRa, C4 cells were transfected with both gRNAs and treated with or without doxycycline for 24 h. The harvested cells were washed once with PBS, fixed with 250 µL of BD Cytofix/Cytoperm solution (BD Bioscience, Franklin Lakes, NJ, USA) for 20 min and permeabilized by washing 2 times in 1X BD Perm/Wash buffer. Post permeabilization, the cells were incubated with pp38 monoclonal antibody (mAb) BD1 at a dilution of 1:500 followed by secondary goat anti-Mouse IgG Alexa Fluor 488 (Invitrogen, Waltham, MA, USA). After the final labelling step, the cells were washed 3 times and then resuspended in PBS. Data were collected using DIVA 8 acquisition software and an LSR Fortessa (BD Biosciences) and analysed in FlowJo (version X10.0.r2, Tree Star, Ashland, OR, USA). A minimum of 10,000 events were collected for each sample. The gating strategy to identify pp38 positive cells is shown in Supplementary Figure S1. In brief, samples were gated on cells (SSC-A vs. FSC-A) and singlets (FSC-A vs. FSC-W) and pp38 expression was identified as cells positive for AF488. C4 cells without doxycycline treatment were used to determine the pp38 threshold.

### 2.6. RNA Extraction and RT-qPCR

Total RNA was extracted from the transfected cells using the Trizol method (Thermo Fisher Scientific, Paisley, UK) according to the manufacturer’s instruction. Then, 250 ng of total RNA was used to synthesise cDNA using The High Capacity RNA-to-cDNA Kit according to the manufacturer’s instructions (Applied Biosystems™, Waltham, MA, USA). Real-time PCR was performed either via Taqman-based PCR [25] or SYBR Green-based PCR [4]. All primer and probe sequences are listed in Table 1. The PCR condition used for Taqman-based PCR was 95 °C for 15 min followed by 40 cycles of 95 °C for 15 s and 60 °C for 1 min. The PCR conditions for SYBR Green-based PCR was 95 °C for 1 min followed by 45 cycles of 95 °C for 15 s and 60 °C for 1 min. All values were normalized to the expression of the endogenous *GAPDH.* The relative expression of the genes was calculated with the calibration of the genes in the un-transfected controls and determined using the arithmetic comparative 2^−∆∆Ct^ method [24]. All qPCR tests were run in triplicate on the ABI 7500 Fast Real-time PCR System (Thermo Fisher Scientific, Paisley, UK).

### 2.7. Reactivation of MDV from pp38/pp24 Activated 4523T Cells

The MDV reactivation was quantified by co-culturing pp38/pp24 activated 4523T with CEF as described previously with minor modification [26]. Briefly, 6 × 10^5^ of CEF cells per well were seeded into a 24-well plate on the day before co-culturing. Then, 10,000 gRNA transfected C4 cells with and without doxycycline treatment were co-cultivated with CEF for 24 h. After removing the C4 cells, the CEF cells were incubated for a further 5 days or until plaques had formed. The CEF monolayers were fixed with acetone/methanol for 5 min. The plaques were labelled with anti-gB mAb HB-3 [27] followed by the secondary antibody Rabbit Anti-Mouse Immunoglobulins/HRP (Agilent, Santa Clara, CA, USA). The plaques were developed using 0.1 M sodium acetate buffer pH 4.8, 3-amino-9-ethylcarbazole substrate solution (AEC, 4 mg/mL) and 30% hydrogen peroxide (Sigma-Aldrich, Burlington, MA, USA). The stained plaques were captured using an EVOS digital microscope (Thermo Fisher Scientific, Waltham, MA, USA) at 20× magnification.

### 2.8. Immunofluorescence Assay (IFA)

The cytotoxicity induced by overexpression of pp38/pp24 in C4 cells was determined with an immunofluorescence assay using confocal microscopy. Cells were coated on the coverslips using Corning™ Cell-Tak (Corning, Deeside, UK) 24 h pt. After being fixed with 4% paraformaldehyde (ChemCruz, Santa Cruz Biotechnology, Inc., Dallas, TX, USA) and permeabilized with 0.1% Triton X-100, the cells were labelled with mAb BD1 to detect pp38 expression followed by Alexa fluor 488 secondary antibody. Cell nuclei were then stained with 4,6-diamidino-2-phenylindole (DAPI). Images were taken using a Leica TCS SP5 confocal laser scanning microscope (Leica Microsystems, Wetzlar, Germany) and analysed using LAS AF Lite software.

### 2.9. Caspase Assay Using Caspase-3/7 Green Reagent

24 h pt, 1 × 10^5^ of gRNA-transfected C4 cells with and without doxycycline treatment were plated into wells of a 96-well plate. Cells were stained with IncuCyte^®^ Caspase-3/7 Green Reagent (Sartorius, Ann Arbor, MI, USA) at 1:1000 dilution for 2 h. The images were captured by label-free high throughput IncuCyte S3 at 4× objective (Sartorius AG, Gottingen, Germany).

### 2.10. DNA Fragmentation Assay

The C4 cells transfected with sgA or pp38/pp24 gRNAs and treated with or without doxycycline were harvested at 24 h pt for DNA fragmentation analysis. The cells were washed twice with ice cold PBS buffer and suspended in 700 µL ice cold lysis buffer (10 mM Tris pH 7.4, 5 mM EDTA and 1% triton X-100) for 20 min on ice. The cells were then centrifuged at 11,000× *g* for 20 min at 4 °C to separate nuclear DNA from the fragmented DNA (supernatant). RNase was added to the supernatant at a concentration of 50 µg/mL and incubated at 37 °C for 30 min followed by the addition of proteinase-K (0.1 mg/mL) and further incubation for 30 min at 37 °C. An equal volume of phenol:chloroform (50:50) was added to the supernatant and vortexed briefly for 30 s. The mixture was centrifuged at 11,000× *g* at 4 °C for 30 min. The aqueous layer was transferred into a fresh tube. A 1/10th volume of 3M sodium acetate solution and an equal volume of 95% ethanol were added and incubated at −20 °C for 2 h followed by centrifugation at 11,000× *g* at 4 °C for 30 min. The pellet was washed once with 75% ethanol and resuspended in 10 µL of TE buffer. The fragmented DNA was analysed on 1.0% agarose gel.

## 3. Results

### 3.1. pp38/pp24 Activation by CRISPRa

MDV can be reactivated from many LCLs via cocultivation with CEF [1]. Varying populations of lytic replication have been observed with different MDV cell lines; this appears to be related to the level of spontaneous virus reactivation (unpublished data). The ability of MDV cell lines to form plaques in CEF allows us to assess the changes in the level of virus reactivation after pp38/pp24 activation by CRISPRa. To this end, we compared the plaque-forming ability of four MDV cell lines (HP8, 4523T, 760S and 226O) in CEF via cocultivation of 10,000 cells with CEF monolayers. Out of the four cell lines, only 4523T cells produced plaques (data not shown) in CEF. 4523T was therefore chosen to study pp38/pp24 activation by CRISPRa in subsequent analysis.

**Figure 1:**
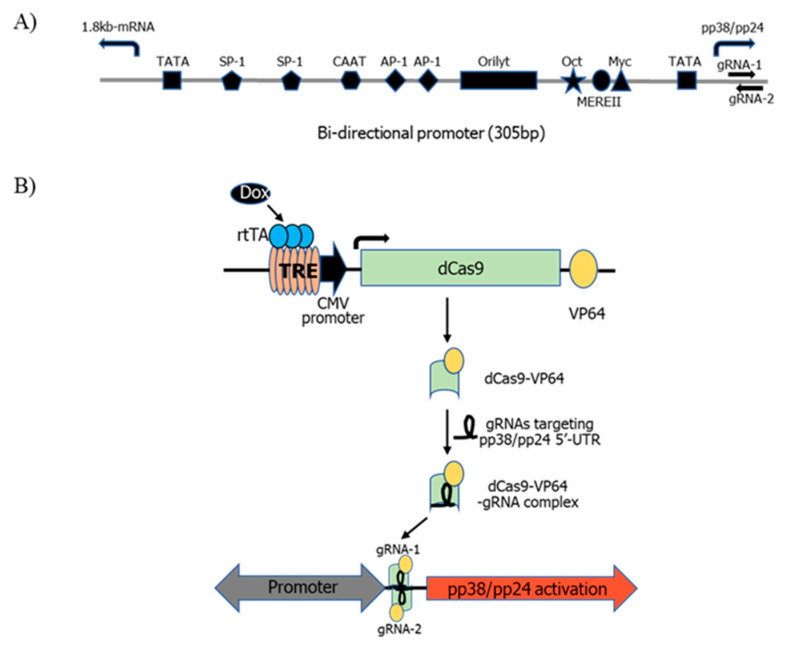
Schematic representation of the pp38/pp24 activation using CRISPRa Tet-On dCas9 VP64 expression system in 4523T cells. (**A**) Schematic representation of the bidirectional promoter with the location of gRNAs for CRISPRa. TATA box (square), SP-1 site (pentagon), CAAT box (haxagon), AP-1 site (diamond), AT-rich replication origin sequence (OriLyt) (rectangle), Oct-1 binding site (star), the Meq recognition sequences MERE II (circle) and Myc binding site (triangle) are indicated. (**B**) CRISPRa Tet-On dCas9 VP64 system for doxycycline-inducible pp38/pp24 activation. 4523T clone C4 expressing dCas9-VP64 were co-transfected with 2 gRNAs. Upon doxycycline (dox+) treatment, rtTA (reverse tetracycline transactivator) binds to the tetracycline response element (TRE) which expresses dCas9-VP64 protein. dCas9-VP64 binds to the gRNAs and forms dCas9-VP64-gRNA complex. The dCas9-VP64-gRNA complex binds to the target site of the pp38/pp24 5′-UTR and activates the pp38/pp24 transgene expression.

To examine the effect of pp38/pp24 on the lytic switch of MDV in a latently infected MDV cell line through activation of pp38/pp24 expression by CRISPRa, we first established a 4523T cell line stably expressing Tet-inducible dCas9-VP64 via transfection of pHAGE-TRE-dCas9-VP64 into 4523T cells followed by geneticin selection and single cell cloning. A purified C4 clone was selected to study pp38/pp24 activation by CRISPRa. It has been reported that the region of −400 to −50 bp upstream from the transcription start site (TSS) is a peak window of active gRNAs for CRISPRa [28]. However, the activity of gRNAs targeting the 5′-UTR of the gene has also been shown to be effective in the CRISPRa system [28,29]. We tested the CRISPRa activity of two high-scoring gRNAs located at the 5′-UTR of pp38/pp24 designed using the gRNA designing tool CRISPOR due to the lack of good quality gRNA candidates at the promoter region. The schematic representation of the bidirectional promoter driving pp38/pp24 and 1.8 kb-mRNA with the location of gRNAs for CRISPRa and CRISPRa Tet-On dCas9 VP64 system for doxycycline-inducible pp38/pp24 activation are illustrated in Figure 1. The two gRNAs targeting the 5′-UTR of pp38/pp24 or a non-specific control gRNA sgA were transfected into C4 cells, which were then treated with or without 1 µg/mL doxycycline for 24 h. Upon doxycycline treatment, a reverse tetracycline-controlled transactivator (rtTA) binds to the TRE thereby activating dCas9-VP64 expression, and the dCas9-VP64-gRNA complex induces targeted activation of pp38/pp24 expression. To measure the level of pp38 protein expression in the transfected cells before and after treatment, cells were labelled with pp38-specific antibody BD1 and analysed using FACS.

**Figure 2:**
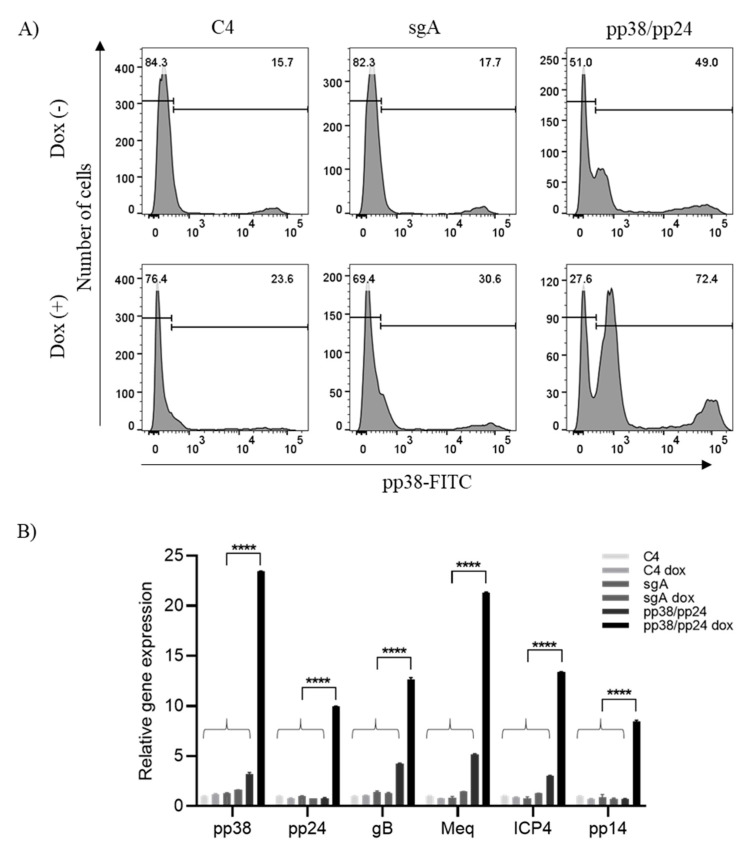
pp38/pp24 overexpression by CRISPRa manoeuvres the expression of other MDV viral genes. (**A**) Quantification of pp38-expressing C4 cells following CRISPRa using flow cytometry. C4 cells were transfected with sgA or pp38/pp24-specific gRNAs in the presence (C4 dox, sgA dox and pp38/pp24 dox) or absence of doxycycline (C4, sgA and pp38/pp24). The cells were incubated with anti-pp38 mAb BD1 followed by Alexa fluor 488 secondary antibody 24 h pt. The labelled cells were subjected to flow cytometry analysis. The percentage of the pp38 –ve and pp38 +ve populations are shown for each population. (**B**) RT-qPCR analysis of MDV viral gene expression in pp38/pp24-overexpressed population (pp38/pp24 dox) and the control cells. C4 cells were transfected with sgA or pp38/pp24-specific gRNAs in presence (C4 dox, sgA dox and pp38/pp24 dox) or absence of doxycycline (C4, sgA and pp38/pp24). RNA was extracted 24 h pt, followed by RT-qPCR analysis using gene specific primers and probes where applicable. The histogram representing the relative gene expression of pp38, pp24, gB, meq, ICP4 and pp14 in C4 cells with different treatments. Expression was normalized to the host gene GAPDH. The relative fold change was compared with C4 cells and expressed as fold change (2^−ΔΔCT^). Asterisk indicates statistically significant differences between pp38/pp24-overexpressing cells and each of the control cells. ****, *p* < 0.0001.

Prior to induction, we detected a similar percentage of cells expressing pp38 in both C4 control cells and sgA-transfected cells (15.7% and 17.7% respectively, Figure 2A). The majority of these pp38 positive cells expressed high level of pp38. Interestingly, C4 cells transfected with the pp38/pp24-specific gRNAs contained two populations of pp38 expressing cells that comprised 49% of the total population (Figure 2A). This increase in the percentage of cells expressing pp38 before induction was not expected but was likely due to leaky expression of the dCas9-VP64. We observed cells that either expressed high levels or low levels of pp38 protein. The difference in the pp38 expression level is likely reflecting the difference of the pp38 level before addition of doxycycline, with a low level of pp38 protein originated from non-pp38-expressing cells and a high level of pp38 originated from existing pp38-expressing cells. The addition of doxycycline treatment induced a moderate increase in the percentage of C4 and sgA-transfected cells expressing pp38 (23.6% and 30.6% respectively). The addition of doxycycline to pp38/pp24 gRNA-transfected cells lead to a significant increase in the percentage of cells positive for pp38 protein expression when compared to the sgA-transfected cells (Figure 2A). Within the low pp38-expressing cell population there was a 3.3-fold increase in the percentage of cells expressing pp38 and a 1.7-fold increase in the level of pp38 expression (as measured via mfi) in the pp38/pp24 gRNA-transfected cells when compared to sgA-transfected cells. In the high pp38-expressing cell population there was a 1.6-fold increase in the percentage of cells expressing pp38 and a 1.2-fold increase in the level of pp38 expression (as measured via mfi) in the pp38/pp24-transfected population when compared with the sgA-transfected cells. These results indicate that a much higher percentage of pp38/pp24 gRNA-transfected cells are expressing a greater amount of pp38 protein when compared to the sgA and C4 control cell lines.

The increase in pp38 expression was also reflected by the pp38 transcript level (Figure 2B). RT-qPCR analysis of the control gRNA sgA-transfected C4 cells with and without doxycycline showed a basal level of pp38/pp24 mRNA expression. However, pp38/pp24-specific gRNA-transfected C4 cells induced with doxycycline showed robust induction with ~24-fold increase of pp38 mRNA and 10-fold increase of pp24 mRNA, compared to only ~3-fold modest increase of pp38 and no change for pp24 observed in the non-induced doxycycline-negative cells.

### 3.2. pp38/pp24 Activation Induces Overexpression of Other MDV Lytic Genes

Having achieved upregulation of pp38/pp24 using CRISPRa, we next analysed the expression of MDV lytic genes ICP4, gB, Meq and pp14 in response to pp38/pp24 overexpression. Measurement of transcript levels reveals that there was significant increase in all of these lytic genes in doxycycline-induced C4 cells overexpressing pp38/pp24 (Figure 2B). The increase in expression of the transcripts measured via RT-qPCR varied from 7-fold (pp14) and 12-fold (ICP4 and gB) to 20-fold (Meq). Untreated pp38/pp24 gRNA-transfected cells showed a marginal 2–4-fold increase in expression of ICP4, gB and Meq transcripts, probably due to the leaky expression of dCas9-VP64, although interestingly, such increases were not observed with pp14 and pp24, which showed a lower level of upregulation with doxycycline treatment compared to the other lytic genes. Control C4 cells not targeted to overexpress pp38/pp24 showed only a background level of expression. These results clearly show that overexpression of pp38/pp24 triggers other lytic genes, promoting the lytic switch, which is in agreement with the previous published data [8].

### 3.3. Differential Expression of Host Genes in Response to pp38/pp24 Overexpression

**Figure 3:**
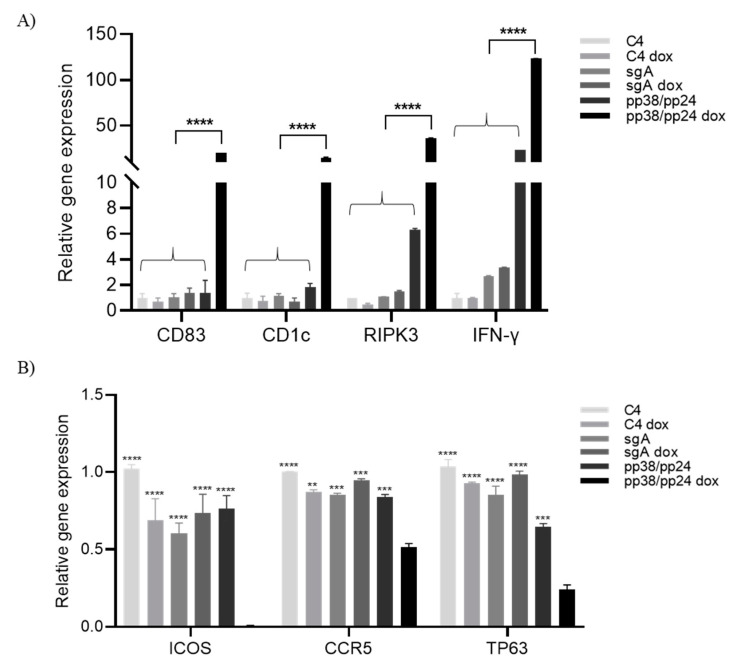
Differential expression of host genes in pp38/pp24-overexpressed C4 cells. RT-qPCR analysis of differential expression of host genes in pp38/pp24-overexpressed population and the control cells. C4 cells were transfected with sgA or pp38/pp24-specific gRNAs in presence (C4 dox, sgA dox and pp38/pp24 dox) or absence of doxycycline (C4, sgA and pp38/pp24). RNA was extracted 24 h pt, followed by RT-qPCR analysis using gene-specific primers. The histogram represents the relative gene expression of host genes in C4 cells. Expression was normalized to the endogenous GAPDH. The relative fold change was compared with C4 cells and expressed as fold change (2^−ΔΔCT^). (**A**) Histogram representing upregulation of host gene (CD83, CD1c, RIPK3 and INF-γ) transcripts in C4 cells. (**B**) Histogram representing downregulation of host gene (CCR5, TP63 and ICOS) transcripts in C4 cells. Asterisk indicates statistically significant differences between pp38/pp24-overexpressing cells and each of the control cells. **, *p* < 0.01; ***, *p* < 0.001; ****, *p* < 0.0001.

William et al. (2017) have previously reported that cellular pathways such as T-cell receptor and B-cell receptor and ICOS-ICOSL signaling were affected during the latent to lytic switch of MDV infection [4]. In order to explore the host pathways affected upon pp38/pp24 activation, we quantified the expression level of the selected upregulated (RIPK3, INF-γ, CD1c and CD83) and downregulated (TP63, CCR5 and ICOS) genes during the latent to lytic switch reported previously [4]. As shown in Figure 3A, gene expression was upregulated by 12-fold for CD1c, 20-fold for CD83, 23-fold for RIPK3 and 120-fold for INF-γ respectively in pp38/pp24-overexpressing cells compared to the control cells. In contrast, a similar level of expression of those genes was observed in most of the control cells except for the moderate increase of RIPK3 and IFN-γ in C4 cells with pp38/pp24 gRNAs but without doxycycline treatment. This has been attributed to leaky pp38 expression. Similarly, although there are different degrees of reduction of TP63, CCR5 and ICOS genes in all the control cells not induced to express pp38/pp24 (C4 dox, sgA, sgA dox and pp38/pp24) compared to un-treated C4 cells, doxycycline-induced pp38/pp24-overexpressing cells showed significant reduction of those genes compared to all control cells (Figure 3B), particularly the expression of ICOS in pp38/pp24-overexpressing cells was undetermined. These results confirm that targeted activation of pp38/pp24 by CRISPRa also induces differential host gene expression, the same set of genes affected during the latent to lytic switch of MDV infection reported previously [4].

### 3.4. Enforced Expression of pp38/pp24 Enhances MDV Reactivation

**Figure 4:**
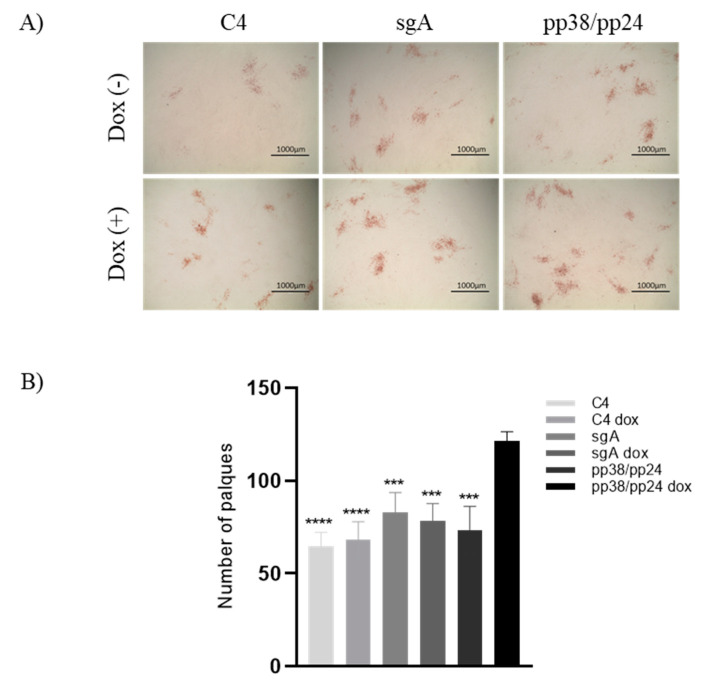
MDV reactivation was enhanced with pp38/pp24 overexpression. (**A**) C4 cells were transfected with gRNAs. At 24 h pt, 1 × 10^4^ C4 cells were co-cultivated with CEFs in 24-well culture plate. At 5 days post co-cultivation, the plaques were labelled with gB mAb (HB3) and anti-mouse HRP conjugate. The stained plaques were captured using the EVOS digital microscope (Thermo Fisher Scientific, Waltham, MA, USA) at 20x magnification. The scale bar, 1000 μm. (**B**) A histogram representing the average number of plaques formed from duplicates of each sample. Asterisk indicates statistically significant differences between pp38/pp24-overexpressing cells and each of the control cells. ***, *p* < 0.001; ****, *p* < 0.0001.

Having demonstrated that the CRISPRa-induced activation of pp38/pp24, in the C4 clone of the 4523T cell line, upregulated other MDV lytic genes and induced differential expression of several host genes as observed previously in the latent to lytic switch, we examined the role of increased pp38/pp24 expression in virus reactivation. To this end, 10,000 cells of CRISPRa-activated C4 cells were co-cultivated with CEF monolayers. The C4 cells transfected with the non-targeting gRNA, sgA, with or without doxycycline treatment and the C4 cells transfected with pp38/pp24 gRNAs but without doxycycline treatment were included as controls. All samples were plated in duplicate. The number of MDV plaques produced in the CEF monolayer after 5 days co-cultivation was enumerated after staining. The average number of plaques from duplicate wells were compared. A similar number of plaques were obtained from the control cells with 65 plaques for C4, 68 for C4 dox, 83 for sgA, 79 for sgA dox and 74 for pp38/pp24 without doxycycline treatment. In contrast, 122 plaques were observed in cells with pp38/pp24 activated through CRISPRa, which is a 1.8-fold increase when compared to the controls, confirming that CRISPRa-induced pp38/pp24 overexpression results in increased virus replication in 4523T cells (Figure 4).

### 3.5. Cytolysis Induced by pp38/pp24 Expression

**Figure 5:**
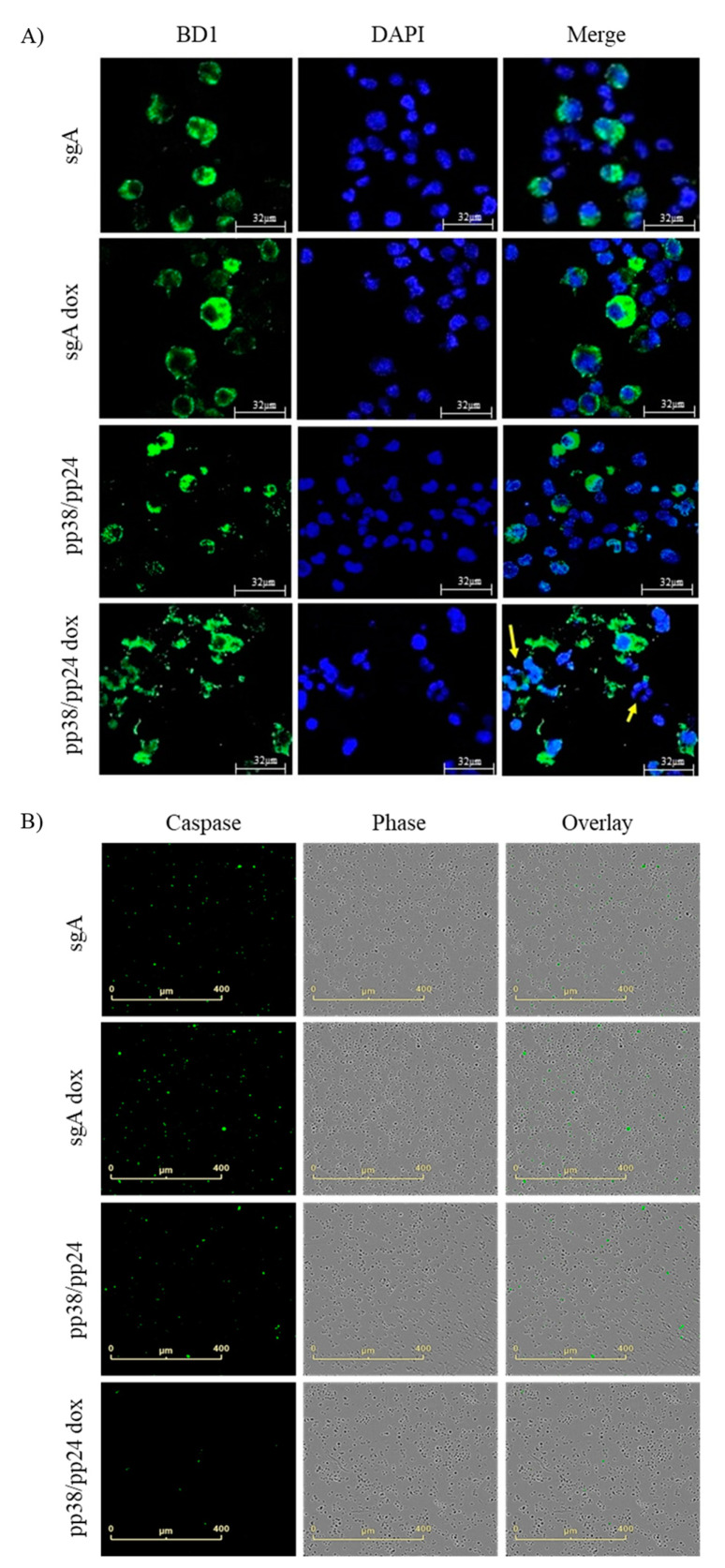
pp38/pp24 overexpression induces increased cytopathic effect but inhibits apoptosis. (**A**) Cytopathic effect in pp38/pp24-overexpressed cells was analysed via IFA. C4 cells transfected with sgA or pp38/pp24-specific gRNAs with and without doxycycline treatment were fixed 24 h pt. The cells were labelled with anti-pp38 mAb BD1 followed by goat anti-mouse AF488 (green) and nuclei were stained with DAPI (blue). The images were captured using confocal image analyser at 10× magnification. sgA, sgA dox and pp38/pp24 (no dox) represent the control cells showing intact nuclei whereas pp38/pp24-overexpressed (pp38/pp24 dox) cells showed necrotic nuclei (yellow arrow). The scale bar, 32 μm. (**B**) Apoptosis assay quantified using Incucyte^®^ Caspase-3/7 Green Reagent. C4 cells transfected with sgA or pp38/pp24-specific gRNAs with or without doxycycline were treated with IncuCyte^®^ Caspase-3/7 Green Reagent. The green fluorescence indicates the apoptotic cells and cells were analysed with the IncuCyte S3 live-cell imaging system. The control cells (sgA, sgA dox and pp38/pp24) showed many green fluorescence cells whereas the pp38/pp24 dox showed few green fluorescence cells. The scale bar, 400 μm.

To study the phenotype of the enforced pp38/pp24-expressing cells, IFA was carried out on the pp38/pp24 gRNAs or control sgA-transfected cells treated with or without doxycycline, using pp38-specific mAb BD1. As shown in Figure 5A, cells transfected with sgA alone, sgA+dox and pp38/pp24 gRNAs showed a normal pattern of pp38 expression in cytoplasm and nuclei were intact. However, pp38/pp24+dox cells showed discrete pp38 expression with many cells showing cytolytic changes and necrotic nuclei, as has been reported previously in pp38-expressing cells [30]. To further explore the cell death pathway in pp38/pp24-overexpressing cells, an apoptosis assay was performed using Caspase 3/7 green reagent and measured with an IncuCyte S3 Live-Cell Image analyser. The apoptotic cells were identified as Caspase 3/7 positive. As seen in Figure 5B, Caspase 3/7 green labelling in sgA-transfected C4 cells with or without doxycycline represented the background level of apoptosis. C4 cells transfected with specific pp38/pp24 gRNAs showed almost no Caspase 3/7 labelling in the presence of doxycycline, indicating strong inhibition of apoptosis. Reduction of Caspase 3/7 labelling in the C4-pp38/pp24 gRNA-transfected cells in the absence of doxycycline is likely due to the ‘leaky’ expression of dCas9-VP64. To further confirm the inhibition of apoptosis by pp38, we carried out a DNA fragmentation assay, a hallmark of apoptosis, on the C4 cells transfected with sgA or pp38/pp24 gRNAs treated with or without doxycycline. As shown in the Supplementary Figure S2, control C4 cells and sgA-transfected C4 cells either in the presence or absence of doxycycline showed characteristic DNA laddering, suggestive of apoptosis. C4 cells transfected with pp38/pp24-specific gRNAs with no doxycycline treatment also showed strong DNA laddering. However, C4 cells transfected with pp38-specific gRNAs with doxycycline treatment showed drastically reduced DNA laddering. The presence of fragmented DNA in these cells and the presence of Caspase 3/7 expression (Figure 5B) are presumably from the un-transfected cell population. Taken together we conclude that pp38/pp24 overexpression in these cells inhibits apoptosis thus aiding cytolytic infection, which is in agreement of the previous finding [14].

## 4. Discussion

The switch between latency and lytic replication is regulated by different factors including epigenetic, transcriptional and post transcriptional changes of viral and host genes [31]. MDV-encoded phosphoprotein pp38 expressed during lytic replication is widely regarded as a biomarker for the latency to lytic switch of MDV infection [9]. Activation of pp38 following induction of the latency to lytic switch of MDV infection in LCLs has been demonstrated using DNA methylation inhibitor 5-Aza-CR or HDAC inhibitor NaB [3]. Both 5-Aza-CR and NaB are drugs with ubiquitous effects on gene expression and are therefore not ideal for determining whether pp38 is a biomarker of lytic replication or if it plays a central role in inducing the lytic switch. In order to address this question, we employed the CRISPRa Tet-On system for targeted overexpression of pp38 in 4523T cells. As pp38 and pp24 share the same promoter and the first 65 amino acids at the N-terminus, targeted activation by targeting the promoter or 5′-UTR region using CRISPRa will have the same effect on both proteins. The CRISPRa Tet-On system has a tightly regulated dCas9-inducible expression with minimal off-target effects. We chose 4523T cells, over other MDV cell lines, due to the presence of a spontaneous lytic switching population which would allow us to study the importance of pp38/pp24 gene expression in the lytic cycle under the favourable niche of the LCL.

MDV pp38 and pp24 are driven from the 305 bp bi-directional promoter located between the transcription start sites of the pp38/pp24 gene and the 1.8-kb gene family regulating the transcription of pp38/pp24 and 1.8-kb mRNA [32,33]. The bi-directional promoter contains two TATA boxes, enhancer motifs and the MDV origin of replication (OriLyt) [11,34]. Although the promoter is the common target site for CRISPRa, gRNAs targeting the 5′-UTR region are also effective for targeted activation of the gene [28,29]. The lack of good quality gRNAs within the bi-directional promoter sequence led to our investigation of two gRNAs at the 5′-UTR of pp38/pp24. Moreover, targeting the 5′-UTR region has the advantage of avoiding any potential disturbance of all active motifs including OriLyt. Indeed, we have demonstrated that CRISPRa using these two gRNAs was successful in inducing targeted overexpression of pp38/pp24 as measured by both FACS analysis and RT-qPCR.

We next examined the expression of MDV lytic genes ICP4, gB, Meq and pp14. The results show that pp38/pp24 overexpression resulted in the upregulation of all these lytic genes although with different fold changes. It has been reported that pp38 plays an important role in regulating the transcriptional activity of the bi-directional promoter [35], and co-expression of pp38 and pp24 is required for this activity [13,36,37]. As one of the transcripts belonging to the 1.8-kb gene family [36], increased expression of pp14 is most likely to be an effect of CRISPRa-induced pp38/pp24 expression. The induction of the latent to lytic switch by pp38/pp24 overexpression has also been reflected by the increased number of MDV plaques when co-cultivated with CEF (Figure 4), indicating a more direct role for pp38/pp24 to enhance the switch to lytic infection. However, pp38/pp24 is not the sole factor required for the latent to lytic switch as the virus can still be activated from LCL MSB-1, containing a pp38/pp24 deletion, when co-cultured with CEF [26]. Previously, Ding et al. tested the potential binding of pp38/pp24 to the promoter using gel mobility shift assay and three overlapping digoxigenin-labelled probes spanning the entire bi-directional promoter. The result showed that pp38/pp24 was a partner in DNA binding and the binding site resides within the 73-bp region which contains the OriLyt, Oct-1, Myc and MEREII sites [35]. Shigekane et al. reported that the bi-directional promoter activity was regulated by a viral or cellular factor(s) induced by MDV infection, and such factor(s) bind to a 30-bp fragment in the promoter region [34]. Interestingly, the 30-bp fragment containing Oct-1, Myc and MEREII sites identified in their study is within the 73-bp region identified by Ding et al. [35]. Whether pp38/pp24 is one of the factors binding to the 30-bp sequence needs further investigation. Based on the data presented in this report, we speculated that pp38/pp24 triggers the latent to lytic switch by binding to the OriLyt sequence of the bi-directional promoter. Further work is needed to verify this.

William et al. showed differential expression of several host genes in response to MDV lytic infection of LCL [4]. In our study involving targeted activation of pp38/pp24, we observed upregulation of host genes such as CD83, CD1c, RIPK3 and IFN-γ (Figure 3A). Upregulation of IFN-γ can have multiple effects on the cellular milieu. For example, IFN-γ upregulates CD83 and CD1c, which can inhibit stimulation of T-cells [38], potentially facilitating MDV replication. This is consistent with the finding that MDV infection of chickens resulted in the upregulation of IFN-γ as early as 3 days post infection, and its level was indicative of increasing MDV loads [39,40]. Overexpression of pp38/pp24 also downregulates host genes TP63, CCR5 and ICOS, which agrees with the report showing that MDV reactivation inhibits T-cell activation via the TCR/ICOS signaling pathway [4].

CRISPRa-induced pp38/pp24 overexpression also triggered morphological changes in the LCL. Confocal imaging of pp38/pp24-overexpressed cells showed scattered nuclei, a characteristic feature of necrotic cells (Figure 5A). To confirm the nature of the cytolytic changes, we examined the status of apoptosis. The observation that pp38/pp24-overexpressing cells showed almost no Caspase 3/7 expression is consistent with the published data of pp38 expression in necrotic lymphomas [30]. IFN-γ upregulation can activate RIPK3 [30], a marker for necroptosis that exhibits morphological features of apoptosis and necrosis [41,42]. Cells undergoing necroptosis do not show the characteristic DNA fragmentation seen in apoptosis [43]. A reduced DNA fragmentation was observed in the pp38/pp24-activated cells indicative of necroptosis (Supplementary Figure S2). Based on the observations of the inhibition of apoptosis (Figure 5B and Supplementary Figure S2) and upregulation of RIPK3 (Figure 3A), we speculate necroptosis as a potential cause of cell death in pp38/pp24-overexpressing cells. However, further studies are required to confirm this.

Taken together, our studies show that the targeted activation of pp38/pp24 can be achieved in MDV-transformed LCLs using robust CRISPRa technology. The targeted overexpression of the MDV gene pp38/pp24 initiated an orchestrated set of events that resulted in the sequential upregulation of a cascade of other MDV lytic genes, ICP4, gB, Meq and pp14, similar to that seen in lytic MDV infection. These data strongly suggest that pp38/pp24 expression is a critical step in triggering the latency to lytic switch, supporting the hypothesis that pp38 expression is more than just a lytic biomarker of MDV infection. An increase in the number of MDV plaques on CEF co-cultivated with pp38/pp24-activated LCLs could be the result of differential regulation of host genes such as IFN-γ that favors MDV replication through various pathways including inhibition of T-cell activation and induction of necroptosis. As both pp38 and pp24 are required for the transactivation activity on the bi-directional promoter, we speculate both proteins are involved in the latent/lytic switch. More investigation is needed to determine whether the switch is induced by pp38/pp24 singly or cooperatively and the potential necroptosis pathway involved in pp38/pp24-overexpressed cells.

## Figures and Tables

**Table 1:** Primers and probes used in real-time PCR.

Target Gene	Primer/Probe	Sequence (5′-3′)
pp38 [25]	pp38-F	GAAAACAGAAGCGGAATGCG
	pp38-R	CGATCCAAAGCGCTCATCTC
	pp38-probe	CCCCGCATTTCTCGCCGTCCTC
Meq [25]	Meq-F	AGCCGGAGAGGC TTTATGC
	Meq-R	GGCCCGAATACA AGGAATCC
	Meq-probe	TTACCGAGGAT CCCGAAC
ICP4 [25]	ICP4-F	CGCCACACGAGAACACAATG
	ICP4-R	GGTTGGAGTAGAGCTGCAACTGT
	ICP4-probe	CGGCCCAGTACAGCCTGCGG
gB [25]	gB-F	GGTTCAACCGTGATCCGTCTA
	gB-R	CGATTCCTTCACCCCACT
	gB-probe	ACCGCCGCGAAAATGTC
pp24	pp24-F	TTCCCGAAAACGCATAGAAG
	pp24-R	TCAGAAATCCCGCAAGAAAG
pp14	pp14-F	TGGCTAAATGCGACTTCGGT
	pp14-R	CGTGCAACAACGCCCAAATA
RIPK3	RIPK3-F	AAGGACGCCTCAACCACATC
	RIPK3-R	GCTGGTCACTGTGGGGTAAG
IFN-γ	IFN-γ-F	AAGGACGCCTCAACCACATC
	IFN-γ-R	GCTGGTCACTGTGGGGTAAG
CD1c [4]	CD1c-F	AGCAGGCTGGTGCAGATGTA
	CD1c-R	TGGCCCTCGTAAGCAATGTC
CD83 [4]	CD83-F	CACCCTGTGCAATGTTTGGA
	CD83-R	CAAAGCATGTCACAGCAACATCT
CCR5 [4]	CCR5-F	CGGTTTAGCGTTACTCTTGATGTAATAA
	CCR5-R	TGGACGTGTTCAGCTGATGAC
TP63 [4]	TP63-F	CCTCTCCATGCCTTCAACGT
	TP63-R	CGTGAAATAATCCACACAGGATGA
ICOS [4]	ICOS-F	ACACTGCTGATTCTTATTCCTTAAGTGA
	ICOS-R	TACATTGCCACTTGAAAGAAACCTA
GAPDH	GAPDH-F	AGAACATCATCCCAGCGTCC
	GAPDH-R	CGGCAGGTCAGGTCAACA

## Supplementary Materials

The following are available online at https://www.mdpi.com/article/10.3390/microorganisms9081681/s1, Figure S1: Flow cytometry gating strategy for measurement of pp38 protein expression in C4 cells, Figure S2: pp38/pp24 overexpression inhibits apoptosis examined via DNA fragmentation assay.

## References

[1] Davison A.J. (2010). Herpesvirus systematics. Vet. Microbiol..

[2] Nicoll M.P., Proença J.T., Efstathiou S. (2012). The molecular basis of herpes simplex virus latency. FEMS Microbiol. Rev..

[3] Brown A.C., Nair V., Allday M.J. (2012). Epigenetic regulation of the latency-associated region of marek’s disease virus in tumor-derived t-cell lines and primary lymphoma. J. Virol..

[4] Mwangi W.N., Vasoya D., Kgosana L.B., Watson M., Nair V. (2017). Differentially expressed genes during spontaneous lytic switch of marek’s disease virus in lymphoblastoid cell lines determined by global gene expression profiling. J. Gen. Virol..

[5] Halford W.P., Kemp C.D., Isler J.A., Davido D.J., Schaffer P.A. (2001). Icp0, icp4, or vp16 expressed from adenovirus vectors induces reactivation of latent herpes simplex virus type 1 in primary cultures of latently infected trigeminal ganglion cells. J. Virol..

[6] Perng G.C., Slanina S.M., Yukht A., Ghiasi H., Nesburn A.B., Wechsler S.L. (2000). The latency-associated transcript gene enhances establishment of herpes simplex virus type 1 latency in rabbits. J. Virol..

[7] Xie Q., Anderson A.S., Morgan R.W. (1996). Marek’s disease virus (mdv) icp4, pp38, and meq genes are involved in the maintenance of transformation of mdcc-msb1 mdv-transformed lymphoblastoid cells. J. Virol..

[8] Parcells M.S., Dienglewicz R.L., Anderson A.S., Morgan R.W. (1999). Recombinant marek’s disease virus (mdv)-derived lymphoblastoid cell lines: Regulation of a marker gene within the context of the mdv genome. J. Virol..

[9] Lupiani B.M., Liao Y., Jin D., Izumiya Y., Reddy S.M., Samal S.K. (2019). Marek’s disease virus. Avian Virology: Current Research and Future Trends.

[10] Chen X.B., Sondermeijer P.J., Velicer L.F. (1992). Identification of a unique marek’s disease virus gene which encodes a 38-kilodalton phosphoprotein and is expressed in both lytically infected cells and latently infected lymphoblastoid tumor cells. J. Virol..

[11] Cui Z.Z., Lee L.F., Liu J.L., Kung H.J. (1991). Structural analysis and transcriptional mapping of the marek’s disease virus gene encoding pp38, an antigen associated with transformed cells. J. Virol..

[12] Hong Y., Coussens P.M. (1994). Identification of an immediate-early gene in the marek’s disease virus long internal repeat region which encodes a unique 14-kilodalton polypeptide. J. Virol..

[13] Ding J., Cui Z., Lee L.F. (2007). Marek’s disease virus unique genes pp38 and pp24 are essential for transactivating the bi-directional promoters for the 1.8 kb mrna transcripts. Virus Genes.

[14] Gimeno I.M., Witter R.L., Hunt H.D., Reddy S.M., Lee L.F., Silva R.F. (2005). The pp38 gene of marek’s disease virus (mdv) is necessary for cytolytic infection of b cells and maintenance of the transformed state but not for cytolytic infection of the feather follicle epithelium and horizontal spread of mdv. J. Virol..

[15] Bhaya D., Davison M., Barrangou R. (2011). Crispr-cas systems in bacteria and archaea: Versatile small rnas for adaptive defense and regulation. Annu. Rev. Genet..

[16] Knott G.J., Doudna J.A. (2018). Crispr-cas guides the future of genetic engineering. Science.

[17] Zhang F. (2019). Development of Crispr-Cas Systems for Genome Editing and Beyond.

[18] Dong C., Fontana J., Patel A., Carothers J.M., Zalatan J.G. (2018). Synthetic crispr-cas gene activators for transcriptional reprogramming in bacteria. Nat. Commun..

[19] Forstneric V., Oven I., Ogorevc J., Lainscek D., Praznik A., Lebar T., Jerala R., Horvat S. (2019). Crispra-mediated foxp3 gene upregulation in mammalian cells. Cell Biosci..

[20] Elbasani E., Falasco F., Gramolelli S., Nurminen V., Gunther T., Weltner J., Balboa D., Grundhoff A., Otonkoski T., Ojala P.M. (2020). Kaposi’s sarcoma-associated herpesvirus reactivation by targeting of a dcas9-based transcription activator to the orf50 promoter. Viruses.

[21] Mwangi W.N., Smith L.P., Baigent S.J., Beal R.K., Nair V., Smith A.L. (2011). Clonal structure of rapid-onset mdv-driven cd4+ lymphomas and responding cd8+ t cells. PLoS Pathog..

[22] Baigent S.J., Ross L.J., Davison T.F. (1996). A flow cytometric method for identifying marek’s disease virus pp38 expression in lymphocyte subpopulations. Avian Pathol..

[23] Zhang Y., Tang N., Sadigh Y., Baigent S., Shen Z., Nair V., Yao Y. (2018). Application of crispr/cas9 gene editing system on mdv-1 genome for the study of gene function. Viruses.

[24] He X., Tan C., Wang F., Wang Y., Zhou R., Cui D., You W., Zhao H., Ren J., Feng B. (2016). Knock-in of large reporter genes in human cells via crispr/cas9-induced homology-dependent and independent DNA repair. Nucleic Acids Res..

[25] Baigent S.J., Petherbridge L.J., Howes K., Smith L.P., Currie R.J., Nair V.K. (2005). Absolute quantitation of marek’s disease virus genome copy number in chicken feather and lymphocyte samples using real-time pcr. J. Virol. Methods.

[26] Zhang Y., Luo J., Tang N., Teng M., Reddy V., Moffat K., Shen Z., Nair V., Yao Y. (2019). Targeted editing of the pp38 gene in marek’s disease virus-transformed cell lines using crispr/cas9 system. Viruses.

[27] Barrow A.D., Burgess S.C., Howes K., Nair V.K. (2003). Monocytosis is associated with the onset of leukocyte and viral infiltration of the brain in chickens infected with the very virulent marek’s disease virus strain c12/130. Avian Pathol..

[28] Gilbert L.A., Horlbeck M.A., Adamson B., Villalta J.E., Chen Y., Whitehead E.H., Guimaraes C., Panning B., Ploegh H.L., Bassik M.C. (2014). Genome-scale crispr-mediated control of gene repression and activation. Cell.

[29] Sanson K.R., Hanna R.E., Hegde M., Donovan K.F., Strand C., Sullender M.E., Vaimberg E.W., Goodale A., Root D.E., Piccioni F. (2018). Optimized libraries for crispr-cas9 genetic screens with multiple modalities. Nat. Commun..

[30] Cho K.O., Ohashi K., Onuma M. (1999). Electron microscopic and immunohistochemical localization of marek’s disease (md) herpesvirus particles in md skin lymphomas. Vet. Pathol..

[31] Kennedy P.G., Rovnak J., Badani H., Cohrs R.J. (2015). A comparison of herpes simplex virus type 1 and varicella-zoster virus latency and reactivation. J. Gen. Virol..

[32] Bradley G., Hayashi M., Lancz G., Tanaka A., Nonoyama M. (1989). Structure of the marek’s disease virus bamhi-h gene family: Genes of putative importance for tumor induction. J. Virol..

[33] Zhu G.S., Iwata A., Gong M., Ueda S., Hirai K. (1994). Marek’s disease virus type 1-specific phosphorylated proteins pp38 and pp24 with common amino acid termini are encoded from the opposite junction regions between the long unique and inverted repeat sequences of viral genome. Virology.

[34] Shigekane H., Kawaguchi Y., Shirakata M., Sakaguchi M., Hirai K. (1999). The bi-directional transcriptional promoters for the latency-relating transcripts of the pp38/pp24 mrnas and the 1.8 kb-mrna in the long inverted repeats of marek’s disease virus serotype 1 DNA are regulated by common promoter-specific enhancers. Arch. Virol..

[35] Ding J., Cui Z., Lee L.F., Cui X., Reddy S.M. (2006). The role of pp38 in regulation of marek’s disease virus bi-directional promoter between pp38 and 1.8-kb mrna. Virus Genes.

[36] Jiabo D., Zhizhong C., Shijin J., Sanjay R. (2006). The enhancement effect of pp38 gene product on the activity of its upstream bi-directional promoter in marek’s disease virus. Sci. China C Life Sci..

[37] Ding J., Cui Z., Jiang S., Li Y. (2008). Study on the structure of heteropolymer pp38/pp24 and its enhancement on the bi-directional promoter upstream of pp38 gene in marek’s disease virus. Sci. China C Life Sci..

[38] Fujimoto Y., Tedder T.F. (2006). Cd83: A regulatory molecule of the immune system with great potential for therapeutic application. J. Med. Dent. Sci..

[39] Kaiser P., Underwood G., Davison F. (2003). Differential cytokine responses following marek’s disease virus infection of chickens differing in resistance to marek’s disease. J. Virol..

[40] Xing Z., Schat K.A. (2000). Expression of cytokine genes in marek’s disease virus-infected chickens and chicken embryo fibroblast cultures. Immunology.

[41] Degterev A., Huang Z., Boyce M., Li Y., Jagtap P., Mizushima N., Cuny G.D., Mitchison T.J., Moskowitz M.A., Yuan J. (2005). Chemical inhibitor of nonapoptotic cell death with therapeutic potential for ischemic brain injury. Nat. Chem. Biol..

[42] Dhuriya Y.K., Sharma D. (2018). Necroptosis: A regulated inflammatory mode of cell death. J. Neuroinflam..

[43] Giampietri C., Starace D., Petrungaro S., Filippini A., Ziparo E. (2014). Necroptosis: Molecular signalling and translational implications. Int. J. Cell Biol..

