# Supplementary material for: CRISPR-Mediated Gene Activation (CRISPRa) of pp38/pp24 Orchestrates Events Triggering Lytic Infection in Marek’s Disease Virus-Transformed Cell Lines
Source: Microorganisms. 2021 Aug 8;9(8):1681. doi: 10.3390/microorganisms9081681 (PMC8398467; doi:10.3390/microorganisms9081681)
Supplement: Supplementary file 1 [file microorganisms-09-01681-s001.zip › microorganisms-1280005-SI.pdf]

microorganisms

Supplementary Materials

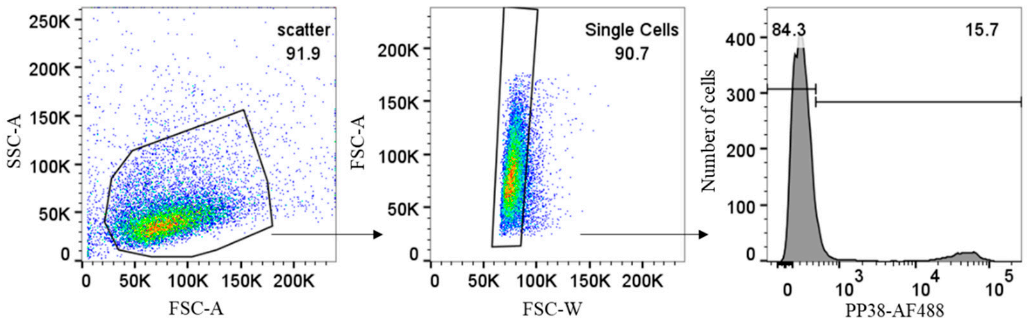

Supplementary Figure S1. Flow cytometry gating strategy for measurement of pp38 protein expression in C4 cells. Cells were transfected with gRNAs and treated with or without doxycycline for 24 h. Cells were harvested, fixed, permeabilised and incubated with a mouse anti-pp38 antibody followed by an Alexa Fluor 488 labelled secondary antibody. Antibody labelling was measured via flow cytometry, using a BD Biosciences LSR Fortessa and DIVA 8 software and analysed with FLOWJO 10.7.2. Cells were first gated on forward scatter area (FSC-A) versus side scatter area (SSC-A) to give population 1 (scatter), which eliminated small debris events, followed by FSC-A versus FSC-W (singlets) to isolate single cells only. A histogram of AF-488 signal (530/30 488nm-A), which corresponds to levels of pp38 protein expression, was then used to quantify the percentage of cells expressing pp38 in the different conditions. The example data shown is that for C4 cells without doxycycline treatment.

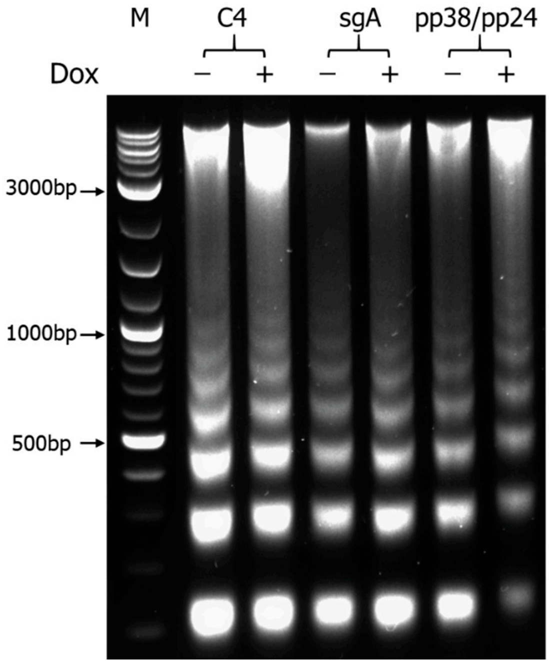

Supplementary Figure S2. pp38/pp24 overexpression inhibits apoptosis examined via DNA fragmentation assay. Cells were transfected with gRNAs and treated with or without doxycycline. C4 cells transfected with sgA or pp38/pp24-specific gRNAs in the presence (C4 dox, sgA dox and pp38/pp24 dox) and absence of doxycycline (C4, sgA and pp38/pp24) were harvested and fragmented DNA were extracted 24 h post transfection. The fragmented DNA were electrophoresed in 1.0% agarose gel.
